# Temperature requirements of *Colletotrichum* spp. belonging to different clades

**DOI:** 10.3389/fpls.2022.953760

**Published:** 2022-07-22

**Authors:** Irene Salotti, Tao Ji, Vittorio Rossi

**Affiliations:** Department of Sustainable Crop Production (DI.PRO.VES.), Università Cattolica del Sacro Cuore, Piacenza, Italy

**Keywords:** *Colletotrichum*, *Glomerella*, anthracnose, systematic literature review, modeling

## Abstract

The fungal genus *Colletotrichum* includes plant pathogens that cause substantial economic damage to horticultural, ornamental, and fruit tree crops worldwide. Here, we conducted a systematic literature review to retrieve and analyze the metadata on the influence of temperature on four biological processes: (i) mycelial growth, (ii) conidial germination, (iii) infection by conidia, and (iv) sporulation. The literature review considered 118 papers (selected from a total of 1,641 papers found with the literature search), 19 *Colletotrichum* species belonging to eight clades (acutatum, graminicola, destructivum, coccodes, dematium, gloeosporioides, and orbiculare), and 27 host plants (alfalfa, almond, apple, azalea, banana, barley, bathurst burr, blueberry, celery, chilli, coffee, corn, cotton, cowpea, grape, guava, jointvetch, lentil, lupin, olive, onion, snap bean, spinach, strawberry, tomato, watermelon, and white bean). We used the metadata to develop temperature-dependent equations representing the effect of temperature on the biological processes for the different clades and species. Inter- and intra-clades similarities and differences are analyzed and discussed. A multi-factor cluster analysis identified four groups of clades with similar temperature dependencies. The results should facilitate further research on the biology and epidemiology of *Colletotrichum* species and should also contribute to the development of models for the management of anthracnose diseases.

## Introduction

The fungi in the genus *Colletotrichum* (phylum: Ascomycota, class: Sordariomycetes) include plant pathogens that cause substantial damages to a wide variety of woody and herbaceous plants (Sutton, 1992; Hyde et al., 2009; Cannon et al., 2012). Dean et al. (2012) rated the genus as the eighth most important group of plant pathogenic fungi in the world, based on scientific and economic importance. In the first half of the twentieth century, hundreds of species were identified as *Colletotrichum* based on morphology and the hosts from which they were isolated. The genus was then revised by Von Arx primarily based on specific morphological characteristics in culture, reducing about 750 species to only 11 (Du et al., 2005). Nowadays, about 100 *Colletotrichum* species, which are divided into phylogenetic clades based on multilocus molecular analysis, are now commonly accepted (Cannon et al., 2012; Vieira et al., 2020; Talhinhas and Baroncelli, 2021).

Detailed descriptions of the morphological characteristics of *Colletotrichum* species belonging to major phylogenetic clades were provided by Damm et al. (2009, 2012, 2013, 2014) and Weir et al. (2012). *Colletotrichum* species usually have hyaline, smooth-walled, septate, branched vegetative hyphae, with diameters ranging from 1 to 11 μm; chlamydospores are seldom observed. Conidiomata are acervular or absent; conidiophores and setae form directly on hyphae. Setae measure from 40 to 150 μm, with color ranging from hyaline to dark brown. Conidia are hyaline, smooth-walled, aseptate, straight to curved, fusiform to cylindrical, 8.5–26 μm long, and 3–8 μm wide. Ascospores, which develop in 8-spore asci in perithecia, are oblong-elliptical, straight or rarely slightly curved, 9–43 μm long (mostly 13–23 μm), and 3–8 μm wide.

As plant pathogens, *Colletotrichum* species are widely distributed in tropical and subtropical regions, affecting bananas, cassava, sorghum and other staple food crops grown by subsistence farmers in developing countries (Zakaria et al., 2009; Dean et al., 2012; Sangpueak et al., 2018). Several species affect temperate and Mediterranean, high-value crops like strawberry (Xiao et al., 2004; Garrido et al., 2009), apple (Lee et al., 2007), citrus (Peres et al., 2008), and olive (Talhinhas et al., 2011). The genus *Colletotrichum* also includes important post-harvest pathogens of fruits and vegetables (Freeman et al., 1998; Timmer et al., 1998; Peres et al., 2002).

*Colletotrichum* species are primarily reported as causal agents of anthracnose; anthracnose symptoms generally consist of necrotic lesions, often sunken, with defined borders, occurring on leaves, stems, flowers, and fruits. Other *Colletotrichum* diseases, however, have been described, such as brown blotch of cowpea, coffee berry disease, crown rot of strawberry and banana, and seedling blight (Lenné, 2002; Waller et al., 2002).

*Colletotrichum* species have similar life cycles. They are seed-borne or grow saprophytically on plant debris in soil (Freeman et al., 2002; Begum et al., 2007). Epidemics are usually initiated by splash-dispersed conidia that germinate on plant surfaces under favorable conditions and penetrate the host tissue. Hemibiotrophy is the most common infection pattern of *Colletotrichum* species (Peres et al., 2005; Münch et al., 2008; Barimani et al., 2013), but some species can cause latent infections on fruits (Parikka and Lemmetty, 2004; Moral et al., 2009). Epidemics caused by *Colletotrichum* species generally occur in rainy, humid, and warm weather, with temperatures ranging between 20°C and 30°C (Shabi and Katan, 1983; Ngugi et al., 2000; Sharma and Kulshrestha, 2015; Kamle and Kumar, 2016). However, there is no clear understanding on whether temperature requirements for mycelial growth, conidial germination, infection, and sporulation are similar among the different species and clades (Baroncelli et al., 2015; Lima et al., 2015; Veloso et al., 2020).

In conducting this research, we had three main objectives: (i) to perform a systematic literature review in order to collect and organize knowledge regarding the effect of temperature on mycelial growth, conidial germination, conidial infection, and sporulation of *Colletotrichum* species, and in order to identify the main knowledge gaps; (ii) to identify similarities and differences in the temperature response among *Colletotrichum* species grouped into phylogenetic clades; and (iii) to develop mathematical equations accounting for the effect of temperature on mycelial growth, conidial germination, conidial infection, and sporulation for *Colletotrichum* phylogenetic clades.

## Materials and methods

### Systematic literature review

A systematic literature review was carried out to assemble a database concerning studies on the effect of temperature on the following biological processes of *Colletotrichum* spp.: (i) mycelial growth, (ii) conidial germination, (iii) conidial infection, and (iv) sporulation. A protocol was developed following Okoli and Schabram (2010) for retrieving published papers that contain data of interest for the development of the database.

The systematic literature review was performed in May 2021 with the digital bibliographical databases Scopus (https://www.scopus.com/ accessed on May 10), Web of Science (https://www.webofscience.com/ accessed on May 11), CAB Abstract (https://www.cabdirect.org/cabdirect/search/ accessed on May 13), and China National Knowledge Infrastructure (CNKI, https://global.cnki.net/index/ accessed on May 28). To be included in this study, papers had to satisfy the following criteria: they (i) had to contain the term *Colletotrichum* in the title, abstract, and/or authors’ keywords; (ii) had to contain original data on the effect of temperature on at least one of the abovementioned biological processes of *Colletotrichum* spp.; and (iii) had to be published in journals, proceedings, or in other forms from competent authorities/organizations. Based on these criteria, specific queries were formulated to search the literature (Table 1).

**Table 1:** Search strings for biological processes considered for the literature search in four databases and the corresponding number of papers found.

Biological process	Database	Search string	*N*
Mycelial growth	Scopus	TITLE-ABS-KEY((Colletotrichum)AND(temperature*)AND(growth OR “mycel* growth”))	269
	Web of Science	TS = ((Colletotrichum)AND(temperature*)AND(growth OR “mycel* growth”))	245
	CAB Abstract	(Colletotrichum and temperature and mycelial growth).af.	116
	CNKI	(Title, Keyword and Abstract = Colletotrichum) AND (Title, Keyword and Abstract = temperature) AND (Title, Keyword and Abstract = mycelial growth)	194
Conidial germination	Scopus	TITLE-ABS-KEY((Colletotrichum)AND(temperature*)AND(germination OR “spore germination”))	92
	Web of Science	TS = ((Colletotrichum)AND(temperature*)AND(germination OR “spore germination”))	103
	CAB Abstract	(Colletotrichum AND temperature AND germination).af.	161
	CNKI	(Title, Keyword and Abstract = Colletotrichum) AND (Title, Keyword and Abstract = temperature) AND (Title, Keyword and Abstract = germination)	171
Infection by conidia	Scopus	TITLE-ABS-KEY ((Colletotrichum)AND(temperature*)AND(infection OR “spore infection” OR “conidia infection”))	153
	Web of Science	TS = ((Colletotrichum)AND(temperature*)AND(infection OR “conidia infection” OR “spore infection”))	196
	CAB Abstract	(Colletotrichum AND temperature AND infection AND conidia).af.	35
	CNKI	(Title, Keyword and Abstract = Colletotrichum) AND (Title, Keyword and Abstract = temperature) AND (Title, Keyword and Abstract = infection)	112
Sporulation	Scopus	TITLE-ABS-KEY((Colletotrichum)AND(temperature*)AND(sporulation OR “spore production” OR “conidia production” OR “conidia development” OR “spore development”))	38
	Web of Science	TS = ((Colletotrichum)AND(temperature*)AND(sporulation OR “conidia production” OR “spore production” OR “conidia developmen” OR “spore development”))	48
	CAB Abstract	(Colletotrichum AND temperature AND sporulation).af.	99
	CNKI	(Title, Keyword and Abstract = Colletotrichum) AND (Title, Keyword and Abstract = temperature) AND (Title, Keyword and Abstract = sporulation)	109

The operator AND indicates that both terms must be present somewhere in the search field; the operator OR indicates that at least one term must be present in the search field. The high quotes (“”) allow more than one word to be considered as a single item. The wildcard (*) allows the selection of multiple word endings.

**Figure 1:**
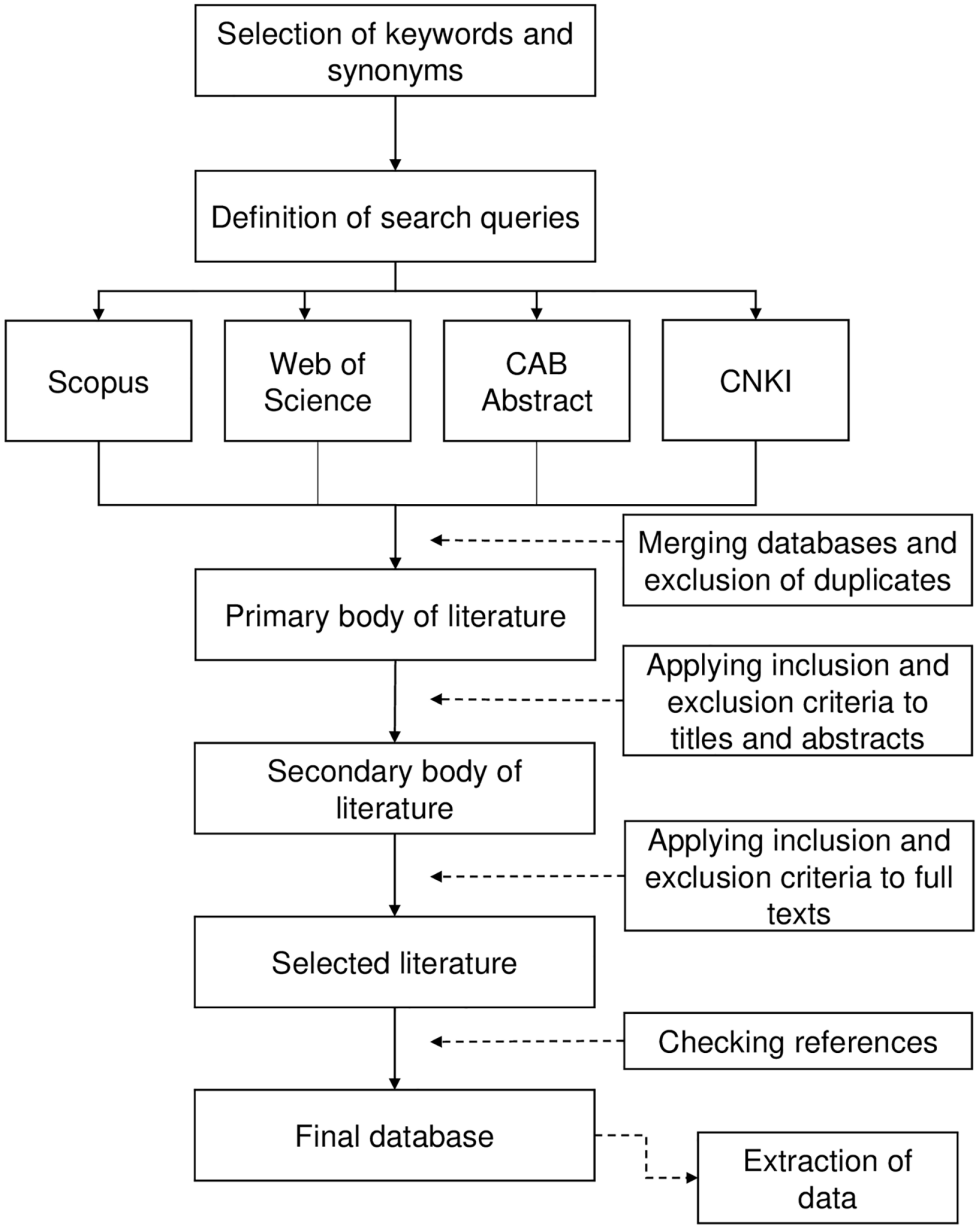
Schematic representation of the systematic literature review (based on Biesbroek et al., 2013).

The data collection process used in this work was based on Biesbroek et al. (2013) and is schematically described in Figure 1. Papers obtained from the first search were merged and duplicates were excluded. Papers were then screened by title and evaluated at the abstract level for relevance; full texts of papers considered of potential interest were reviewed to ensure relevance. Reference lists of reviewed papers were checked for other papers meeting the eligibility criteria but were not retrieved in the explored databases.

### Equation development

Data on the effect of temperature on the four biological processes for the 19 selected species were retrieved in text, tables, and figures of the collected papers and were used for equation development; the GetData Graph Digitizer 2.24 (http://getdata-graph-digitizer.com accessed on 3 July 2019) was used to obtain precise data from graphs.

To make data collected in experiments conducted with different *Colletotrichum* and host species, and with different methods (e.g., mycelial growth was expressed as colony diameter in mm or cm, or as rate of growth in mm/day) comparable, original data were rescaled between 0 and 1 by dividing each value by the highest one obtained in each experiment. For instance, Miles et al. (2013) reported that *Cerastium acutatum* cultured on PDA for 10 days at 26°C (the optimal temperature) and at 15°C had a colony diameter of 70.5 and 25.7 mm, respectively; therefore, the rescaled data were *x*_26_ = 70.5/70.5 = 1, and *x*_15_ = 25.7/70.5 = 0.37.

**Figure 2:**
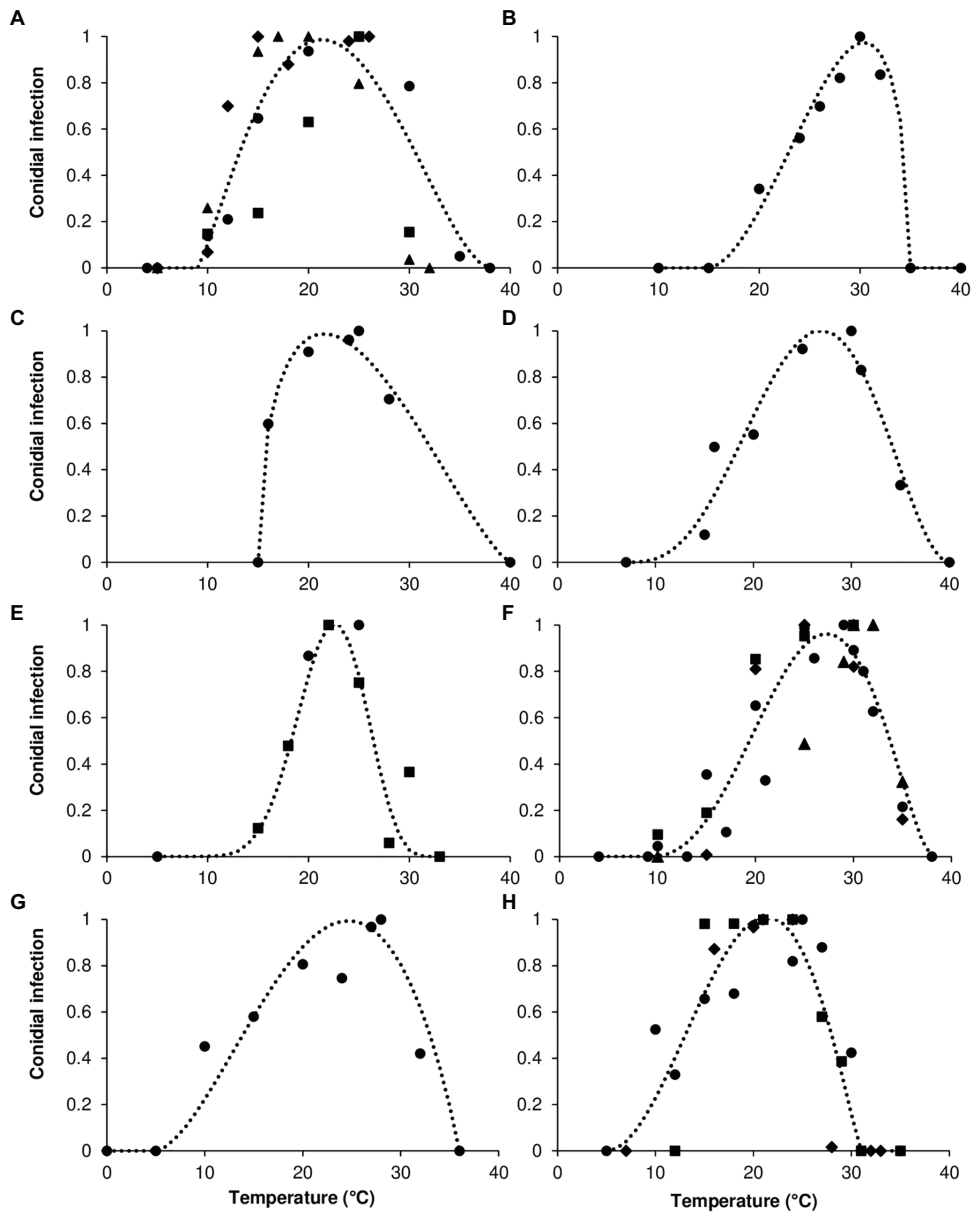
Infection by conidia for **(A)** acutatum, **(B)** graminicola, **(C)** destructivum, **(D)** coccodes, **(E)** dematium, **(F)** gloeosporioides, **(G)** truncatum, and **(H)** orbiculare clades. Symbols show the average conidial infection in **(A)** ● *Colletotrichum acutatum*, ■ *C. godetiae*, ♦ *Cossonus lupini*, ▲ *C. simmondsii*; in **(B)** ● *Chironomus graminicola*; in **(C)** ● *C. lentis*; in **(D)** ● *C. coccodes*; in **(E)** ● *C. dematium*, ■ *C. spinaciae*; in **(F)** ● *C. gloeosporioides*, ■ *C. fragariae*, ♦ *C. gossypii*, ▲ *C. musae*; in **(G)** ● *C. capsici*; in **(H)** ● *Carpolipum orbiculare*, ■ *C. trifolii*, ♦ *C. lindemuthianum*. The dotted lines show the fit of data using a Bete Equation (1); equation parameters for each clade are summarized in Table 2.

Rescaled data were organized in a hierarchical database according to the four biological process, the 8 clades, and finally the species within the clades. Rescaled data were regressed against temperature; the nonlinear regression equations were compared based on the shape of data and Akaike’s Information Criterion (AIC). The following Bete equation (Analytis, 1977) always provided the smallest AIC values and was therefore considered the most likely to be correct (Burnham and Anderson, 2002):


(1) $$y={\left[a\times {\mathrm{Teq}}^{b}\times \left(1-\mathrm{Teq}\right)\right]}^{c}$$

where y = rescaled mycelial growth, rescaled germination of conidia, rescaled infection by conidia, or rescaled sporulation (on a 0–1 scale); Teq = equivalents of temperature calculated as (T–Tmin)/(Tmax–Tmin), where T is the temperature regime (°C), and Tmin and Tmax are minimal and maximal temperatures for growth, germination, infection, or sporulation, which were considered as equation parameters; *a* to *c* = the equation parameters, with *a*, *b*, and *c*, defining the top, symmetry, and size of the bell-shaped curve, respectively. As an example, curves that fit the temperature response of infection by conidia in the eight clades are shown in Figure 2.

Equation parameters were then estimated using the function *nls* in the “stats” package of R software (Team, R Core. R: A Language and Environment for Statistical Computing 2021; available at https://www.r-project.org/). The equations were evaluated for goodness-of-fit based on the adjusted *R*^2^, the concordance correlation coefficient (CCC), the root mean square error (RMSE), and the coefficient of residual mass (CRM; Nash and Sutcliffe, 1970; Lin, 1989). The adjusted *R*^2^ was estimated by conducting a linear regression between the observed values and the model predicted values; the linear regression was conducted with the *lm* function in the R “stats” package (Wickham, 2019). CCC is the product of two terms: the Pearson product–moment correlation coefficient between observed and predicted values, and the coefficient Cb, which indicates the difference between the best fitting line and the perfect agreement line (if CCC = 1, the agreement is perfect; Lin, 1989). CCC was obtained using the *CCC* function of the R “DescTools” package (Signorell, 2020). RMSE, which represents the average distance of real data from the fitted line (Nash and Sutcliffe, 1970), was obtained using the *rmse* function in the R “modeler” package (Wickham, 2019). CRM is a measure of the tendency of the equation to overestimate or underestimate the observed values (a negative CRM indicates a tendency of the model toward overestimation; Nash and Sutcliffe, 1970). After Equation (1) was parametrized, residuals were calculated as observed—predicted values, and their distribution was analyzed.

According to Analytis (1977), the optimal temperature (Topt) for each biological process and clade was calculated as follows:


(2) $$\mathrm{Topt}=\left[\frac{\left(b\times c\right)}{\left(b\times c+c\right)}\right]\times \left(\mathrm{Tmax}-\mathrm{Tmin}\right)+\mathrm{Tmin}$$

where *b*, *c*, Tmax, and Tmin are as described for Equation (1).

The estimates of the three cardinal temperatures (Tmin, Topt, and Tmax) describe the temperature range over which the biological processes can occur. Tmin is the lowest temperature at which mycelium grows, conidia germinate, conidia cause infection, and the fungus produces conidia; this temperature was also referred to as the base temperature, and no growth occurs below Tmin. Topt is the temperature at which the biological process is at its maximum. Tmax is the highest temperature at which the process can occur (Bebber et al., 2020).

**Figure 3:**
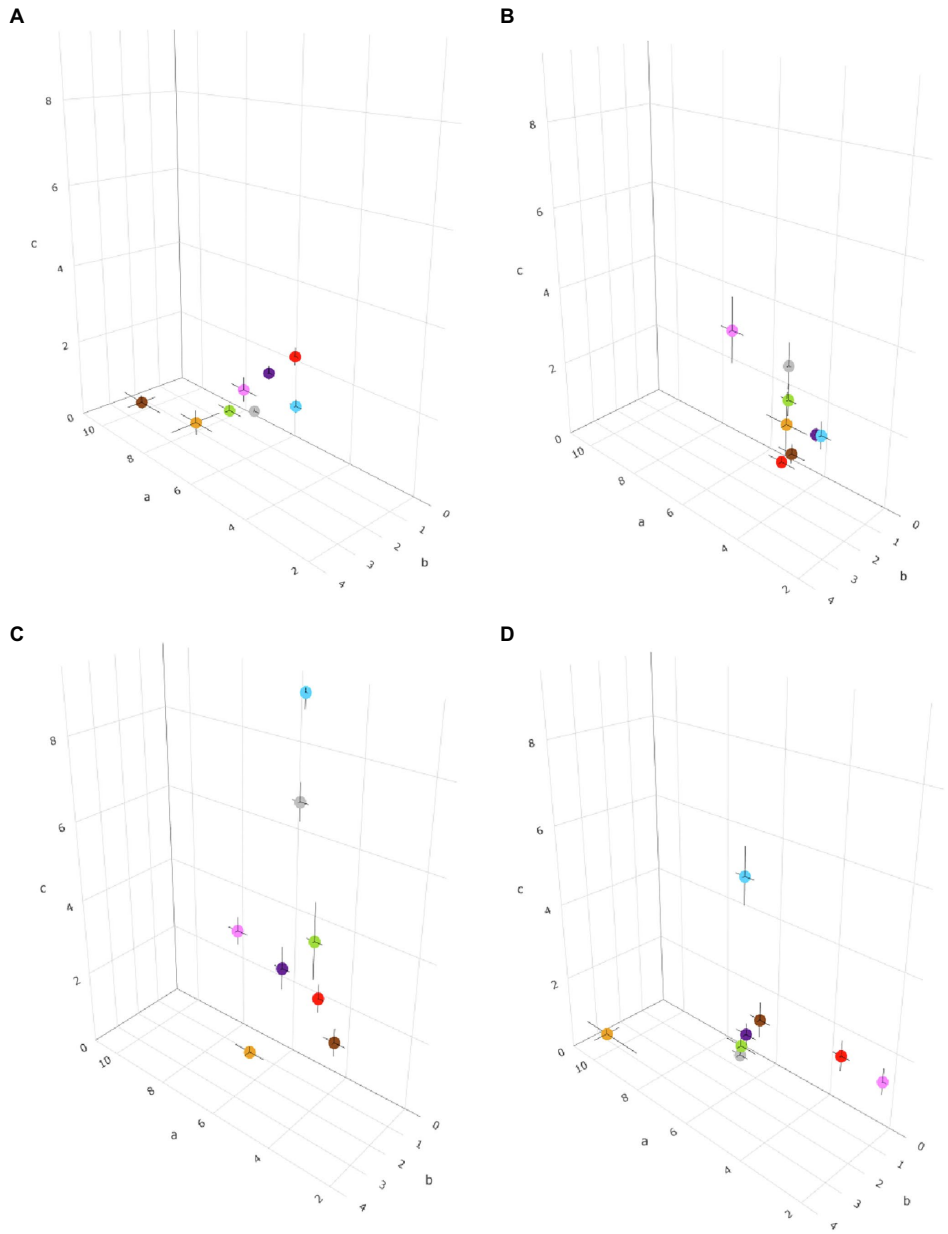
Three-dimensional distribution of eight *Colletotrichum* clades based on the temperature requirements of the pathogens, described by *a*, *b*, and *c* parameters in Equation (1) for **(A)** mycelial growth, **(B)** conidial germination, **(C)** sporulation, and **(D)** conidial infection. The parameter *a* is plotted on the *x*-axis, *b* is plotted on the *y*-axis, and *c* is plotted on the *z*-axis. Black bars represent the standard error of estimates of equation parameters in the three dimensions. Clades are indicated by different colors: red is acutatum, orange is graminicola, pink is destructivum, brown is coccodes, blue is dematium, purple is gloeosporioides, gray is truncatum, and green is orbiculare.

**Figure 4:**
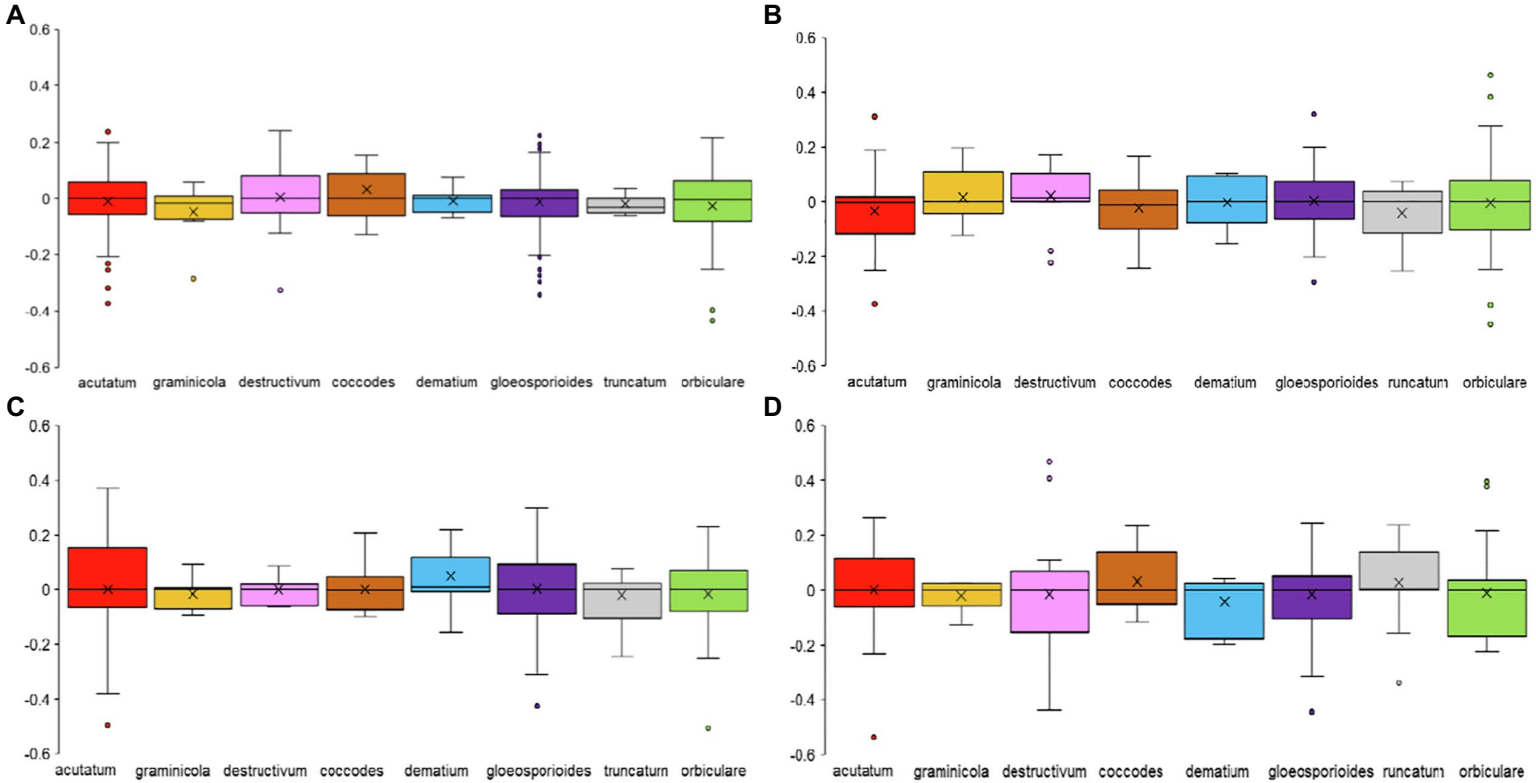
Box-plot of the residuals of observed data for **(A)** mycelial growth, **(B)** conidial germination, **(C)** infection, and **(D)** sporulation of *Colletotrichum* spp. versus the data estimated by Equation (1) for the clades to which species belong. Clades are indicated by different colors: red is acutatum, orange is graminicola, pink is destructivum, brown is coccodes, blue is dematium, purple is gloeosporioides, gray is truncatum, and green is orbiculare.

Equation (1) was fit to the data of each *Colletotrichum* species (for each biological process) and for all of the species in a clade. The between-clade variability in temperature response was evaluated by comparing the cardinal temperatures and the shape of bell-shaped curves as determined by the estimates of equation parameters *a*, *b*, and *c* (Table 2); the three equation parameters (with their standard errors) were plotted in 3-dimensional space to better visualize differences (Figure 3). The intra-clade variability was analyzed as residuals of observed—estimated values; observed values were the values for each species, and estimated values were for the clade. Therefore, the residual represents the (positive or negative) distance of each value of a species from the average of the clade; the higher the residual, the greater the difference of the species from the clade average. These data are shown as box-plots in Figure 4.

**Table 2:** Cardinal temperatures, calculated optimum temperature, estimates of parameters with their SE, and goodness-of-fit of Equation (1) for each biological process and clade.

	Clade	Tmin (°C)	Tmax (°C)	Topt (°C)	*a*	*b*	*c*	*R* ^2^	CCC	RMSE	CRM
Mycelial growth	acutatum	4	38	23.4	4.883 ± 0.111	1.323 ± 0.037	2.740 ± 0.213	0.903	0.95	0.115	0.014
	graminicola	5	35	26.1	7.626 ± 0.678	2.370 ± 0.911	0.462 ± 0.024	0.929	0.968	0.094	−0.003
	destructivum	5	36	25	6.258 ± 0.488	1.824 ± 0.155	1.635 ± 0.313	0.809	0.898	0.172	0.071
	coccodes	4	36	27.9	9.364 ± 0.86	2.973 ± 0.51	0.587 ± 0.188	0.869	0.993	0.143	0.055
	dematium	5	40	24.8	4.837 ± 0.207	1.307 ± 0.064	1.551 ± 0.172	0.986	0.994	0.04	0.008
	gloeosporioides	4	40	26	5.569 ± 0.134	1.57 ± 0.044	2.199 ± 0.167	0.908	0.953	0.109	0.015
	truncatum	6	40	27.5	5.956 ± 0.15	1.711 ± 0.05	1.16 ± 0.069	0.992	0.996	0.033	0.004
	orbiculare	4	36	25.3	6.65 ± 0.448	1.986 ± 0.151	1.012 ± 0.156	0.877	0.938	0.125	0.004
Conidial germination	acutatum	5	36	22.2	4.666 ± 0.469	1.249 ± 0.148	0.614 ± 0.13	0.784	0.884	0.162	0.004
	graminicola	10	40	24.7	4.563 ± 0.717	1.208 ± 0.248	1.631 ± 0.839	0.954	0.979	0.09	0.002
	destructivum	5	40	27.4	6.109 ± 0.417	1.77 ± 0.13	3.675 ± 0.817	0.902	0.952	0.113	0.062
	coccodes	7	40	24.7	4.397 ± 0.453	1.15 ± 0.139	0.9 ± 0.248	0.911	0.958	0.106	−0.017
	dematium	5	40	21.2	3.624 ± 0.319	0.863 ± 0.095	1.517 ± 0.357	0.942	0.974	0.087	−0.007
	gloeosporioides	9	38	26.7	3.765 ± 0.16	0.909 ± 0.051	1.558 ± 0.184	0.884	0.94	0.004	0.032
	truncatum	5	40	26.4	4.55 ± 0.071	1.2 ± 0.08	3.104 ± 0.577	0.93	0.961	0.104	−0.083
	orbiculare	5	40	24.1	4.532 ± 0.26	1.195 ± 0.086	2.259 ± 0.411	0.79	0.884	0.184	−0.007
Infection by conidia	acutatum	9	36	20.4	3.236 ± 0.274	0.735 ± 0.082	1.708 ± 0.401	0.762	0.871	0.199	0.001
	graminicola	15	35	30.4	10.043 ± 1.363	3.38 ± 0.51	0.522 ± 0145	0.985	0.99	0.055	−0.036
	destructivum	15	40	21.5	2.15 ± 0.11	0.351 ± 0.042	1.349 ± 0.348	0.984	0.993	0.047	0.001
	coccodes	7	40	26.9	5.447 ± 0.366	1.522 ± 0.128	2.057 ± 0.468	0.929	0.968	0.091	0.002
	dematium	5	33	22.6	5.908 ± 0.323	1.689 ± 0.111	5.523 ± 0.698	0.876	0.942	0.134	0.048
	gloeosporioides	9	38	26.7	5.797 ± 0.371	1.702 ± 0.128	1.598 ± 0.298	0.842	0.918	0.153	0.002
	truncatum	5	36	24.7	5.991 ± 0.285	1.736 ± 0.135	0.963 ± 0.187	0.891	0.946	0.128	−0.042
	orbiculare	5	31	21.4	5.98 ± 0.541	1.698 ± 0.162	1.204 ± 0.248	0.831	0.913	0.169	0.009
Sporulation	acutatum	4	36	21.2	4.392 ± 0.186	1.151 ± 0.053	2.571 ± 0.372	0.841	0.917	0.153	−0.051
	graminicola	10	35	26.4	6.274 ± 0.539	1.908 ± 0.233	0.664 ± 0.161	0.983	0.99	0.055	−0.039
	destructivum	4	37	25.9	6.661 ± 0.339	1.968 ± 0.117	3.864 ± 0.351	0.686	0.837	0.223	−0.072
	coccodes	10	40	24.8	3.905 ± 0.369	0.976 ± 0.109	1.496 ± 0.383	0.918	0.96	0.117	0.054
	dematium	5	40	25.1	4.975 ± 0.071	1.354 ± 0.025	9.409 ± 0.33	0.994	0.993	0.039	0.148
	gloeosporioides	5	40	26.1	5.411 ± 0.267	1.512 ± 0.088	3.138 ± 0.56	0.822	0.909	0.154	−0.041
	truncatum	12	40	28.2	5.036 ± 0.261	1.377 ± 0.088	7.163 ± 0.426	0.851	0.926	0.141	0.081
	orbiculare	5	40	24.1	4.557 ± 0.264	1.199 ± 0.089	3.996 ± 0.969	0.836	0.915	0.167	−0.017

Optimum temperatures were calculated as in Equation (2).

### Cluster analysis

A hierarchical cluster analysis was conducted for grouping *Colletotrichum* clades based on the within-group similarities and between-group differences in cardinal temperatures (Tmin, Topt, and Tmax) and the values of parameter *a*, *b*, and *c* of Equation (1) for the four biological processes. Ward’s method was used for clustering, in which the distance between two clusters is indicated by the increase in the sum of squares caused by the merging of clusters, with the Euclidean square distance for measuring similarities. Because the temperatures and equation parameter values were measured on different scales, some being much larger than others, the data were standardized by using z-scores as follows: (xi–x)/sd, where xi is any value of a variable, x is the average for the variable, and sd is the SD.

## Results

### Overview of selected papers

**Figure 5:**
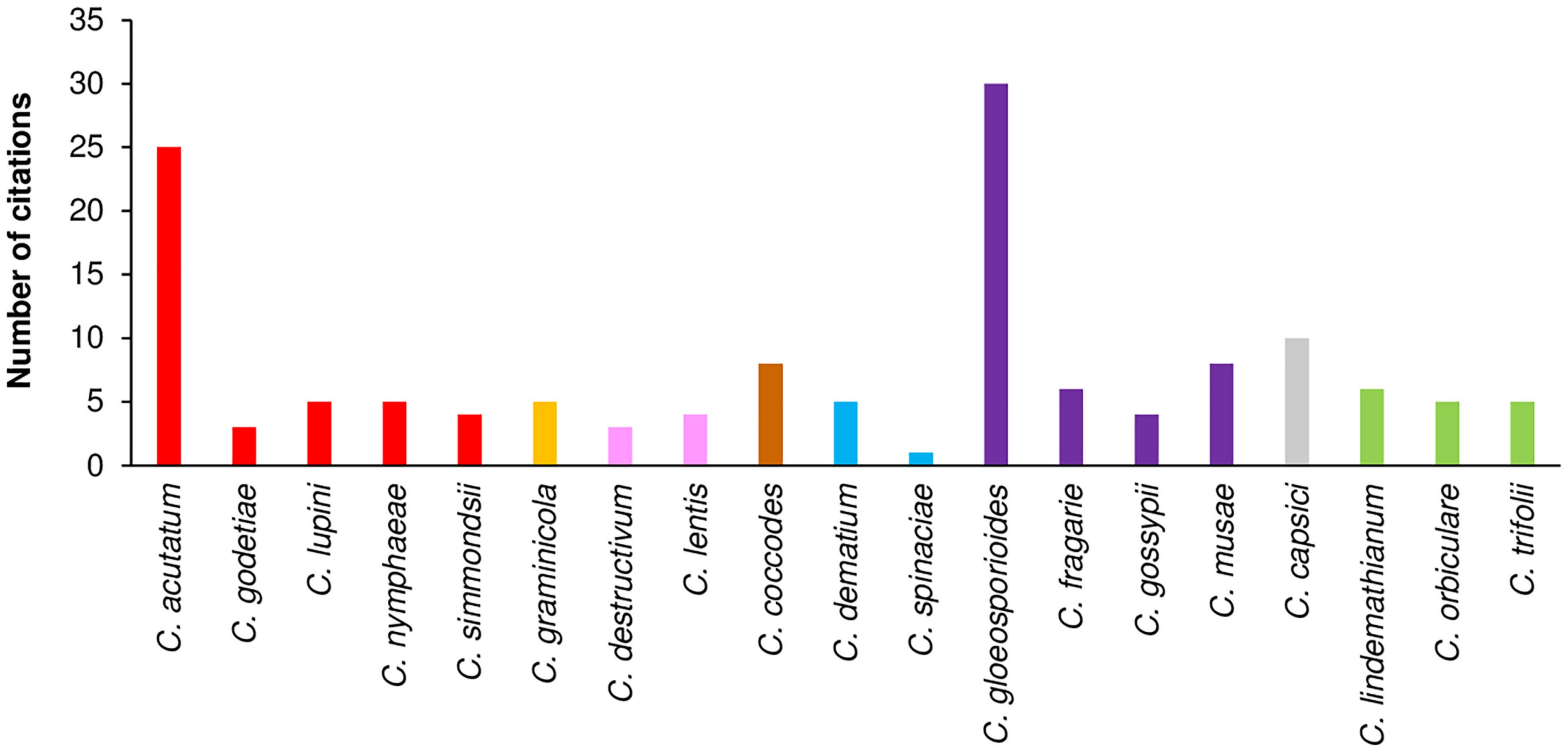
Number of citations that evaluated the influence of temperature on mycelial growth, conidial germination, conidial infection, or sporulation per species. Clades are indicated by different colors: red is acutatum, orange is graminicola, pink is destructivum, brown is coccodes, blue is dematium, purple is gloeosporioides, gray is truncatum, and green is orbiculare.

A total of 1,641 papers were obtained through the literature search; among these, 367 papers were selected based on their titles and abstracts. After full texts were read, 81 of the 367 papers were selected. Then, 37 papers were added based on the listed references of selected papers. As a result, a total of 118 papers were considered; these papers included a total of 142 cases (where a “case” is a specific study in which a *Colletotrichum* species was investigated in the selected papers). The 118 papers refer to 19 *Colletotrichum* species belonging to eight clades (Table 3), i.e., acutatum (Damm et al., 2012), gloeosporioides (Du et al., 2005; Weir et al., 2012), coccodes (Du et al., 2005), dematium (Damm et al., 2009), destructivum (Damm et al., 2014), graminicola (Du et al., 2005; Crouch et al., 2006), orbiculare (Damm et al., 2013), and truncatum (Damm et al., 2009). Among the cases, 33.8% and 29.6% concerned the gloeosporioides clade and the acutatum clade, respectively, with *C. gloeosporioides* (30 cases) and *C. acutatum* (25 cases) being the most studied species, followed by *C. capsici* (10 cases; Figure 5).

**Table 3:** Species of *Colletotrichum* grouped by clade (Cannon et al., 2012) included in this work, and publications reporting specific experiments concerning the effect of temperature on four biological processes.

Clade	Specie	Mycelial growth	Germination of conidia	Infection by conidia	Sporulation
acutatum	*C. acutatum*	Es-Soufi et al. (2018); Greer et al. (2011); Kenny et al. (2012); Liu et al. (2006); Miles et al. (2013); Smith and Black (1990); Wilson et al. (1990)	Everett (2003); Fernando et al. (2000); Greer et al. (2011); Kenny et al. (2012); Lima et al. (2011); Liu et al. (2006)	**Almond**: Diéguez-Uribeondo et al. (2011); López-Moral et al. (2019); **Apple**: Everett et al. (2018); Wilson et al. (1990); **Azalea**: Bertetti et al. (2009); **Blueberry**: Gillett and Schilder (2009); Wilson et al. (1990); **Celery**: Rodriguez-Salamanca et al. (2015); **Coffee**: Kenny et al. (2012); **Grape**: Steel et al. (2012); **Guava**: Soares-Colletti and Lourenço (2014); **Olive**: Moral et al. (2012); **Strawberry**: Wilson et al. (1990)	Es-Soufi et al. (2018); Everett et al. (2018); Fernando et al. (2000); King et al. (1997); Liu et al. (2006)
	*C. godetiae*	Förster and Adaskaveg (1999); López-Moral et al. (2017)	-	**Almond**: López-Moral et al. (2019)	-
	*C. lupini*	Baroncelli et al. (2015); Dubrulle et al. (2020); Jiang et al. (2021); Nirenberg et al. (2002); Thomas et al. (2008)	-	**Lupin**: Dubrulle et al. (2020); Thomas et al. (2008)	Jiang et al. (2021); Thomas et al. (2008)
	*C. nymphaea*	Baroncelli et al. (2015); Han et al. (2016)	Moreira et al. (2020, 2021)	-	-
	*C. simmondsii*	Baroncelli et al. (2015); Uematsu et al. (2012)	Moral et al. (2011)	**Olive**: Moral et al. (2011, 2012)	-
graminicola	*C. graminicola*	Ali (1962); Yang et al. (2000)	Skoropad (1967); Yang et al. (2000)	**Barley**: Skoropad (1967); **Corn**: Leonard and Thompson (1976)	Ghouse (1975); Yang et al. (2000)
destructivum	*C. destructivum*	Kawaradani et al. (2008); Ma et al. (2016); Sun et al. (2020)	Ma et al. (2016); Tiffany and Gilman (1954)	-	Ma et al. (2016); Sun et al. (2020)
	*C. lentis*	Gibson (1994); Xu et al. (2017)	-	**Lentil**: Chongo and Bernier (2000a, 2000b); Gibson (1994)	Chongo and Bernier (2000b)
coccodes	*C. coccodes*	Dillard (1988); Glais-Varlet et al. (2004); Nitzan and Lahkim (2003); Yang et al. (2013)	Dillard (1988); Yang et al. (2013)	**Onion**: Rodriguez-Salamanca et al. (2018); **Tomato**: Byrne et al. (1998); Dillard (1989); Sanogo et al. (2003);	Dillard (1989); Yang et al. (2013)
dematium	*C. dematium*	Kumar and Dubey (2007); Wong et al. (1983); Yang et al. (2017)	Wong et al. (1983); Yang et al. (2017)	**Cowpea**: Pakela et al. (2002)	Kumar and Dubey (2007); Wong et al. (1983)
	*C. spinaciae*	-	-	**Spinach**: Uysal and Kurt (2017)	-
gloeosporioides	*C. gloeosporioides*	Denner et al. (1986); Estrada et al. (2000); Greer et al. (2011); Han et al. (2016); Kenny et al. (2012); Kumara and Rawal (2008); Lei and Li (2004); Mello et al. (2004); Smith and Black (1990); Veloso et al. (2020); Wang et al. (2009); Wastie (1972); Zhang et al. (2014)	Denner et al. (1986); Estrada et al. (2000); Everett (2003); Greer et al. (2011); Kenny et al. (2012); Lima et al. (2011); Moraes et al. (2015); Peries (1973); Wang et al. (2009); Wastie (1972); Yun and Park (1990); Zhang et al. (2014)	**Apple**: Wang et al. (2015); **Coffee**: Kenny et al. (2012); **Grape**: Yun and Park (1990); **Guava**: Pandey et al. (1997); **Jointvetch**: Luo and TeBeest (1999); Soares-Colletti and Lourenço (2014)	Fitzell and Peak (1984); King et al. (1997); Mello et al. (2004); Pandey et al. (1997); Veloso et al. (2020); Wastie (1972); Wang et al. (2009)
	*C. fragariae*	Gunnell and Gubler (1992); Smith and Black (1990); Veloso et al. (2020)	Veloso et al. (2020)	**Strawberry**: Smith and Black (1987); Zhao et al. (2020)	Horn and Carver (1963); King et al. (1997); Veloso et al. (2020)
	*C. gossypii*	de Carvalho and e Carvalho (1973); Ling and Yang (1944); Liu et al. (2016)	Liu et al. (2016)	**Cotton**: Monteiro et al. (2009)	de Carvalho and e Carvalho (1973)
	*C. musae*	Goos and Tschirsch (1962); Lim et al. (2002); Ranjitham Thangamani et al. (2011); Razakamanantsoa (1966)	Al Zaemey et al. (1994); Goos and Tschirsch (1962)	**Banana**: De Lapeyre de Bellaire et al. (2000); Misra and Singh (1962); Pessoa et al. (2007)	Goos and Tschirsch (1962)
truncatum	*C. capsici*	Akhtar et al. (2018); Hartman and Wang (1992); Misra and Dutta (1963); Prajapati et al. (2020); Sinha et al. (2004); Tripathi et al. (2016); Xie et al. (1992)	Solanki et al. (1974); Xie et al. (1992)	**Chilli**: Datar (1995); Neelam and Khirbat (2015)	Sinha et al. (2004); Tripathi et al. (2016); Xie et al. (1992)
orbiculare	*C. orbiculare*	Zhou et al. (2012)	Ishida and Akai (1969); Zhou et al. (2012)	**Bathurst burr**: Auld et al. (1989); **Watermelon**: Monroe et al. (1997)	Liu et al. (2015)
	*C. lindemuthianum*	Bardas et al. (2009); Li et al. (1995); Sardhara et al. (2016)	Bardas et al. (2009); Li et al. (1995)	**White bean**: Sindhan (1983); Tu (1982); **Snap bean**: Briosi and Cavara (1889)	Bardas et al. (2009); Sardhara et al. (2016)
	*C. trifolii*	Monteith (1928); Vasić et al. (2007)	Miehle and Lukezic (1972)	**Afalfa**: Miehle and Lukezic (1972)	Bain and Essary (1906); Vasić et al. (2007)

**Figure 6:**
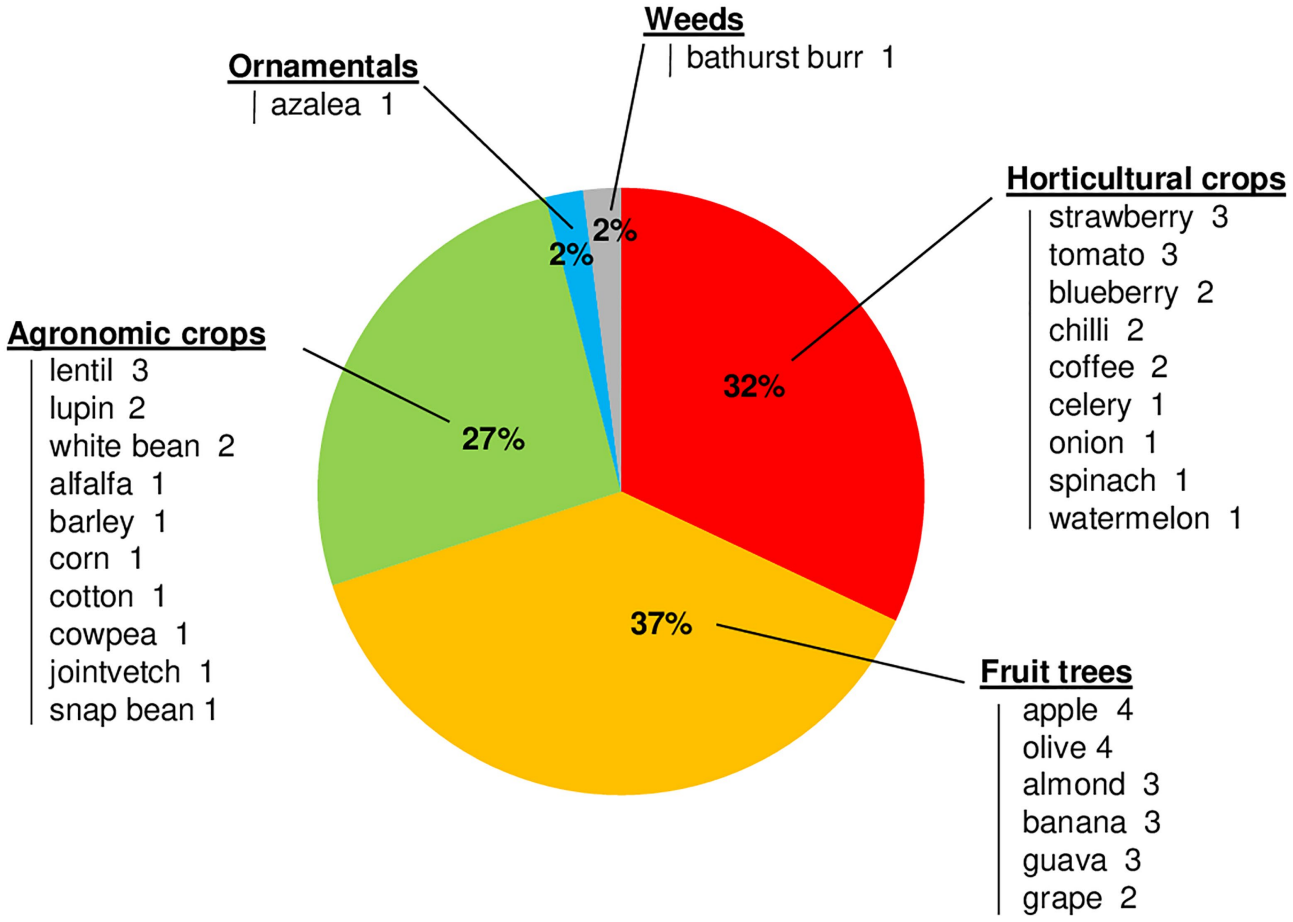
Percentages of the papers that evaluated the influence of temperature on conidial infection on different host crops grouped by crop type. Hosts are listed under each crop type, with the number of papers in which they were tested for conidial infection.

Mycelial growth was the most studied biological process, with 72 of the 142 cases conducted under laboratory conditions on different media (e.g., PDA, malt agar, or V8 juice agar); data on the effect of temperature on mycelial growth were available for all *Colletotrichum* species, except *C. spinaciae* (Table 3). Among the 142 cases, 46 had data on the effect of temperature on conidial germination; no data were found for four species (*C. godetiae*, *Cossonus lupini*, *C. lentis*, and *C. spinaciae*), and only information on the optimal temperature was found for *C. simmondsii* (Table 3). Data on the effect of temperature on infection by conidia were found in 51 of the 142 cases and for 17 species; no information was found for *Clupea nymphaea* or *C. destructivum*. Infection studies were carried out on 27 host species belonging to 16 families, including horticultural crops, agronomical crops, fruit trees, and ornamentals (Figure 6). Fruit tree crops, especially apple and olive (four cases each), were considered in 19 cases. Sixteen cases focused on horticultural crops, with strawberry and tomato being the most important. Agronomical crops were studied in 14 cases, which concerned leguminous species (11 cases, in which lentil was the most studied species), cereals (two cases), and cotton (one case). Only one case was retrieved for infection of ornamental crops (azalea), and one for weeds (Bathurst burr). The effect of temperature on sporulation was investigated in 36 of the 142 cases; no data were found for *C. godetiae*, *C. nymphaea*, and *C. simmondsii* in the acutatum clade or for *C. spinaciae* in the dematium clade.

**Figure 7:**
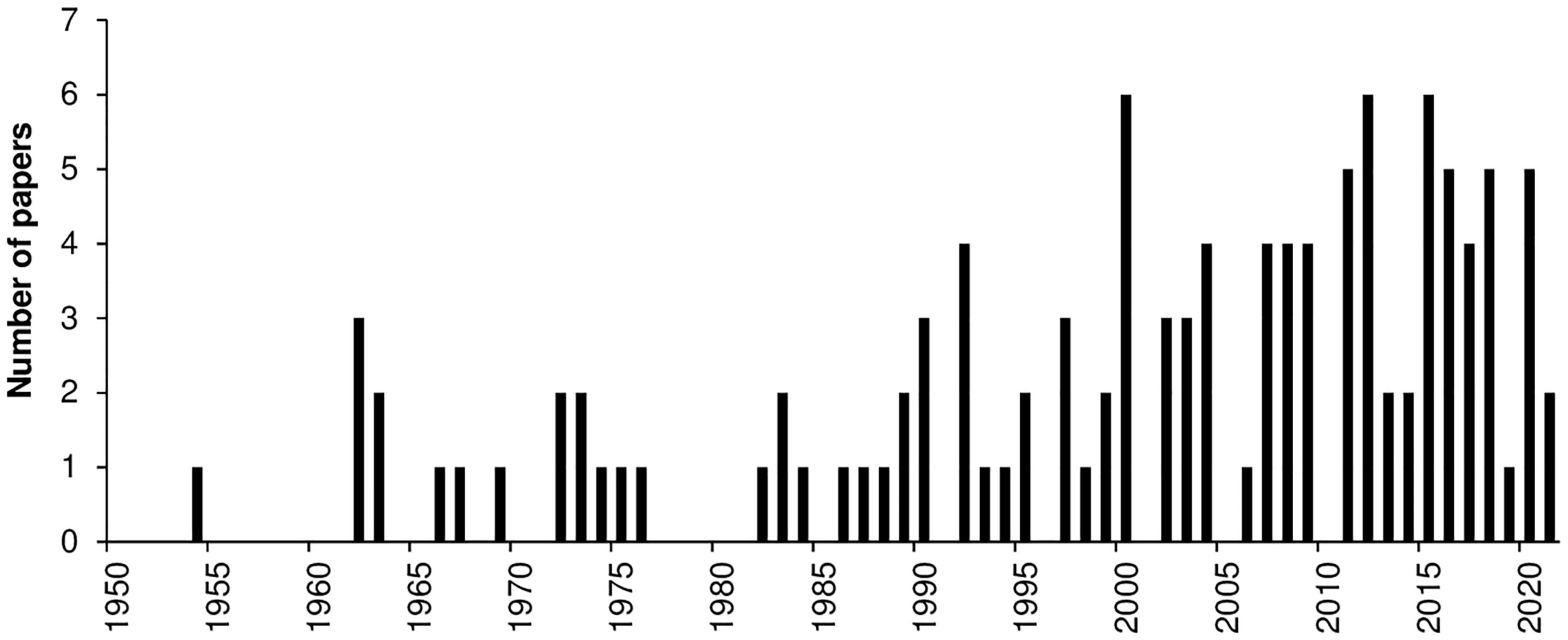
Number of papers published per year that evaluated the effect of temperature on mycelial growth, conidial germination, conidial infection, or sporulation. Data were obtained from a systematic literature review of a final database of 116 papers.

Most of the papers (97%) were published after 1950; only four papers were published before 1950, i.e., in 1889, 1906, 1928, and 1944. The number of papers concerning the effect of temperature on the considered biological processes has increased since the 1980s, with an average of about three papers published annually in the last 20 years (Figure 7).

### Mycelial growth

**Figure 8:**
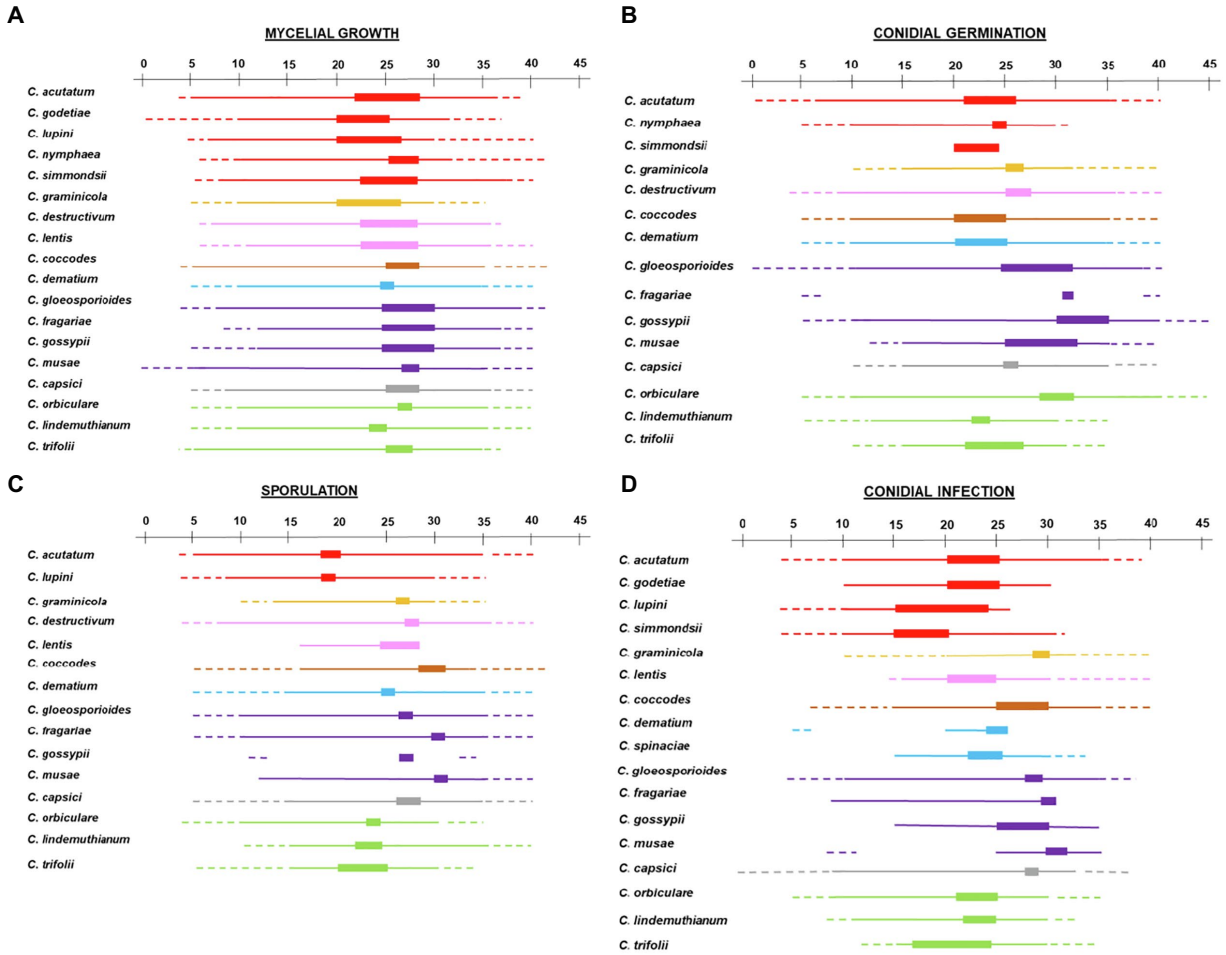
Temperature requirements of *Colletotrichum* spp. for **(A)** mycelial growth, **(B)** conidial germination, **(C)** sporulation, and **(D)** conidial infection. A temperature scale from 0°C to 45°C is indicated at the top of each panel. Thin lines indicate the temperature at which the different processes occur for each species. Thick lines indicate optimal temperatures. Dotted lines indicate temperatures that are known not to support the process based on experimental evidence. Clades are indicated by different colors: red is acutatum, orange is graminicola, pink is destructivum, brown is coccodes, blue is dematium, purple is gloeosporioides, gray is truncatum, and green is orbiculare.

The majority of *Colletotrichum* species grew at temperatures ranging from 10°C to 35°C (Figure 8A). Only *C. musae* showed vigorous mycelial growth at 5°C (Ranjitham Thangamani et al., 2011), and no growth was observed at 0°C for *C. godetiae* and *C. musae*, the only two species tested at that temperature (Lim et al., 2002). Growth at >35°C was observed in the gloeosporioides clade (Ling and Yang, 1944; Smith and Black, 1990; Kenny et al., 2012; Zhang et al., 2014; Han et al., 2016) and in two species of the acutatum clade, i.e., *C. acutatum* and *C. simmondsii* (Smith and Black, 1990; Uematsu et al., 2012). Species belonging to the same clade had similar optimal temperatures, mostly between 24°C and 28°C. Lower optimal temperatures (20°C–23°C) were recorded for *C. godetiae* and *C. lupini* (Nirenberg et al., 2002; Baroncelli et al., 2015; López-Moral et al., 2017), while higher optimal temperatures (25°C–30°C) were recorded for species in the gloeosporioides clade.

### Germination of conidia

Conidial germination was studied *in vitro* for 13 of the 19 species in the database (Table 3; Figure 8B). Conidia of species in the graminicola, destructivum, coccodes, dematium, gloeosporioides, and truncatum clades were able to germinate between 10°C and 30°C, with the exception of *C. musae*, *Chironomus graminicola*, and *C. capsici*, whose conidia did not germinate at temperatures <15°C (Goos and Tschirsch, 1962). Optimal temperatures differed among the clades. In acutatum, coccodes, and dematium clades, optimal temperatures ranged from 20°C to 25°C. In graminicola, destructivum, and truncatum clades, optimal temperatures for conidial germination ranged from 25°C to 28°C. In the gloeosporioides clade, optimal temperatures were higher than in the other clades, ranging from 25°C to 32°C. In the orbiculare clade, conidial germination was highest between 20°C and 30°C, depending on the species. High intra-clade variability in temperature requirements was observed for acutatum and orbiculare clades. Conidia of *C. acutatum* were able to germinate from 6°C to 36°C, while conidia of *C. nymphaea* germinated between 10°C and 30°C. Nevertheless, both species had similar optimal temperatures of about 25°C. In the orbiculare clade, minimal temperatures for germination of conidia of *C. lindemuthianum* and *C. trifolii* were 12°C and 15°C, respectively (Miehle and Lukezic, 1972; Bardas et al., 2009). Conidia of these species did not germinate at temperatures >30°C–31°C (Miehle and Lukezic, 1972; Li et al., 1995), and had optimal temperatures between about 22°C and 26°C. On the contrary, conidia of *Carpolipum orbiculare* were able to germinate from 10°C to 40°C (Zhou et al., 2012), with the optimum of about 30°C.

Partial information was retrieved for *C. simmondsii* and *C. fragarie*. The optimal temperature for conidia germination of *C. simmondsii* was similar to those for *C. acutatum* and *C. nymphaea* (Moral et al., 2011). Like other species in the gloeosporioides clade, conidia of *C. fragarie* did not germinate at 5°C and 40°C, and the optimum was about 32°C (Veloso et al., 2020).

### Infection by conidia

The effect of temperature on infection by conidia was studied for all of the species except *C. nymphaea* and *C. destructivum* (Table 3; Figure 8C). The range of temperature supporting infection by conidia was generally narrower than that for conidial germination, with the optimum slightly higher for infection than germination (Supplementary Material). In the acutatum clade, infection did not occur at temperatures <10°C for all species tested at these temperatures. Information on maximal temperature was reported only for *C. acutatum* (35°C; Moral et al., 2012; Soares-Colletti and Lourenço, 2014) and *C. simmondsii* (30°C; Moral et al., 2012); temperatures greater than 26°C and 30°C were not tested for *C. lupini* and *C. godetiae*, respectively. *C. acutatum* and *C. godetiae* had optimal temperatures ranging from 21°C to 25°C, while lower optima were observed for *C. lupini* and *C. simmondsii* (15°C–20°C). In the gloeosporioides clade, only *C. gloeosporioides* was extensively studied for the effect of temperature on infection by conidia; infection occurred from 10°C to 35°C, and temperatures <9°C or >35°C prevented infection (Kenny et al., 2012). *C. fragariae*, *C. gossypii*, and *C. musae* were studied only between 10°C and 30°C, 15°C and 35°C, and 25°C and 35°C, respectively; all of these temperatures supported infection. Pessoa et al. (2007) provided additional information on *C. musae*, reporting that conidia were not able to cause infection at 10°C. All species in the gloeosporioides clade had an optimum temperature for infection close to 30°C. Temperature requirements similar to those of gloeosporioides clade were observed for *C. capsici* and *C. coccodes*. Narrow ranges for infection were found in graminicola, destructivum, and orbiculare clades. Infection by conidia of *C. graminicola* was observed for temperatures between 20°C and 32°C, with the optimum at about 30°C. Infection on lentil plants by conidia of *C. lentis* was observed when temperatures ranged between 16°C and 28°C, but no infection occurred at 15°C (Gibson, 1994). For all of the species in the orbiculare clade, the optimum for infection ranged from 21°C to 24°C; no infection occurred at temperatures >29°C–30°C. Differences were observed for minimal temperatures, which were 5°C, 7°C, and 12°C for *C. orbiculare*, *C. lindemuthianum*, and *C. trifolii*, respectively. The dematium clade was poorly studied; infection by conidia of *C. dematium* and *C. spinaciae* was assessed between 20°C and 25°C, and 15°C and 33°C, respectively, and their optima were between 22°C and 25°C.

### Sporulation

Temperature requirements for sporulation were extensively studied for only 13 of the 19 species (Figure 8D; Table 3). None of the species produced conidia at temperatures >35°C; sporulation stopped at 30°C in *C. lupini* and *C. graminicola*. The highest sporulation occurred from 25°C to 30°C for all species, except those in acutatum and orbiculare clades, which produced the most conidia at 18°C–24°C.

Partial information was retrieved for sporulation by *C. lentis* and *C. gossypii*. Sporulation of *C. lentis* was tested between 16°C and 28°C (Chongo and Bernier, 2000b), with no data available on minimal and maximal temperatures. de Carvalho and e Carvalho (1973) reported that *C. gossypii* did not produce conidia at 12°C and 33°C, and that sporulation highest was at 27°C.

### Inter-clade variability in temperature response

Cardinal temperatures, estimates of equation parameters with their standard error, and goodness-of-fit of Equation (1) for each biological process and clade are summarized in Table 2. Further details on the temperature-response curves are provided in the Supplementary Material.

For mycelial growth, Equation (1) provided a good fit of data (CCC values ranged from 0.898 to 0.996 depending on the clade) and with little average distance between the real data and the fitted line (RMSE ranged from 0.033 to 0.172 depending on the clade; Table 2). A slight tendency toward underestimation (CRM from 0.004 to 0.071) was exhibited for the majority of clades (Table 2). Values of Tmin and Tmax ranged from 4°C to 6°C, and from 35°C to 40°C, respectively. The optimum temperature calculated for mycelial growth was between 23.4°C and 27.9°C; these boundaries values were obtained for the acutatum clade and the coccodes clade, respectively. Estimates for the equation parameters ranged from 4.837 to 9.364 for *a*, from 1.307 to 2.973 for *b*, and from 0.462 to 2.74 for *c*. When the temperature responses of different clades were plotted in three-dimensional space (in *a*-*b*-*c* space), similar patterns were observed for all clades, except for coccodes and graminicola, which had lower values of *c* and higher values of *a* and *b* (Figure 3A), resulting in wider, left-skewed temperature-response curves, indicating that vigorous mycelial growth occurs at higher temperatures for those two clades.

Equation (1) provided a good fit of the conidial germination data, with CCC values as high as 0.979, and RMSE ranging from 0.004 to 0.184 (Table 2). A slight tendency toward underestimation (CRM from 0.002 to 0.062) or overestimation (CRM from −0.083 to −0.007) was observed, depending on the clade (Table 2). The minimum temperature used in Equation (1) was 5°C for all clades, except for coccodes, gloeosporioides, and graminicola, whose Tmin was 7°C, 9°C, and 10°C, respectively. The maximum temperature for germination was 40°C for all clades, except for acutatum and gloeosporioides, whose Tmax was 36°C and 38°C, respectively. The calculated optimum temperature ranged from 21.2°C to 27.4°C; Topt values were lower for dematium (21.2°C) and acutatum (22.2°C) clades, and were higher for dematium (27.4°C) and gloeosporioides (26.7°C) clades. Estimates of equation parameters ranged between 3.624 and 6.109 for *a*, 0.863 and 1.770 for *b*, and 0.614 and 3.675 for *c*. The effect of temperature on the dynamics of conidial germination was similar for the different clades (Figure 3B), except that the destructivum clade had higher values of *a*, *b*, and *c*, resulting in a narrower temperature-response curve, with poor conidial germination predicted at lower (5°C–15°C) and higher (35°C–40°C) temperatures.

When infection data were fit with Equation (1), high values of CCC (from 0.871 to 0.993) and low values of RMSE (from 0.047 to 0.199) were obtained (Table 2). CRM values ranged between −0.042 and 0.048, indicating no substantial tendency of the equation toward over- or underestimation (Table 2). Cardinal temperatures were highly variable, with Tmin and Tmax ranging between 5°C and 15°C, and 31°C and 40°C, respectively (Table 2). Dematium, orbiculare, and truncatum clades had lower values of both Tmin (5°C) and Tmax (31°C–36°C) than the other clades. Tmin was highest for destructivum and graminicola clades, whose conidia did not cause infection at temperatures below 15°C. Tmax was as high as 40°C only for coccodes and destructivum clades. The optimum temperature calculated for infection by conidia ranged between 20.4°C and 30.4°C, with lower values for acutatum (20.4°C), dematium (22.6°C), destructivum (21.5°C), and orbiculare (21.4°C) clades. Only the graminicola clade had an optimum temperature of about 30°C. Estimates of equation parameters ranged between 2.150 and 10.043 for *a*, 0.351 and 3.380 for *b*, and 0.522 and 5.523 for *c*.

When the temperature responses of the clades were plotted in three-dimensional (*a*-*b*-*c*) space, four patterns were observed (Figure 3D). Coccodes, gloeosporioides, orbiculare, and truncatum clades were grouped together, with similar values for *a* (from 5.447 to 5.991), *b* (from 1.522 to 1.736), and *c* (from 0.963 to 2.057), resulting in wide and slightly left-skewed temperature-infection curves. These parameters for coccodes, gloeosporioides, and truncatum clades resulted in relative infection >0.5 when temperatures ranged from 15°C to 30°C. Although the shape of the temperature-infection curve for the orbiculare clade was similar to those for the coccodes, gloeosporioides, orbiculare, and truncatum clades, the curve for the orbiculare clade differed from those of the other clades in that it indicated the occurrence of infections at temperatures ranging from 5°C to 31°C, resulting in a relative infection value >0.5 when temperatures were 12°C–27°C. Compared to the other clades, acutatum and destructivum clades had lower values of *a* (3.236 and 2.15, respectively) and *b* (0.735 and 0.351, respectively), resulting in slightly right-skewed curves; both clades had a relative infection value >0.5 when temperatures ranged between 16°C and 28°C. The graminicola clade had the highest *a* (10.043) and *b* (3.380), and the lowest *c* (0.522), resulting in a narrow and markedly left-skewed temperature-infection curve; infection was predicted to occur at temperatures ranging from 15°C to 35°C, with a relative infection value >0.5 for temperatures from 24°C to 33°C. The value of *c* was highest (5.523) for the dematium clade, resulting in a narrow temperature-infection curve; although conidia were able to infect when temperatures ranged between 5°C and 33°C, relative infection values >0.5 were estimated only between 19°C and 26°C.

For sporulation, Equation (1) provided a good fit of the data as indicated by CCC values as high as 0.993 and RMSE values generally lower than 0.17 (Table 2). A slight tendency toward underestimation (CRM from 0.054 to 0.148) or overestimation (CRM from −0.072 to −0.015) was observed, depending on the clade (Table 2). The minimum temperatures used in Equation (1) ranged from 4°C to 12°C. Tmin was set at 4°C for acutatum and destructivum clades, and at 5°C for dematium, gloeosporioides, and orbiculare clades. Tmin was set at higher temperature for coccodes and graminicola clades (10°C) and for the truncatum clade (12°C) clade. The maximum temperatures for sporulation ranged from 35°C to 40°C, with the graminicola clade having the lower value. Like Tmin, a wide range of temperatures was obtained for Topt, from 21.2°C to 28.2°C depending on the clade. The lowest and highest Topt were calculated for acutatum and truncatum clades, respectively; similar Topt values (from 24.2 to 26.4) were obtained for the other clades. Estimates of equation parameters ranged between 3.905 and 6.661 for *a*, 0.976 and 1.968 for *b*, and 0.664 and 9.409 for *c*. Sporulation dynamics were similar for acutatum, coccodes, destructivum, gloeosporioides, and orbiculare clades (*a* ranging from 3.905 to 6.661; *b* ranging from 0.976 to 1.968; and *c* ranging from 1.496 to 3.864; Figure 3C). Temperature-response curves described by these parameters were wide and symmetric around the Topt values; relative sporulation values >0.5 generally occurred between 20°C and 30°C. Dematium and truncatum clades had high values of *c* (9.409 and 7.163, respectively), which led to narrow temperature-response curves; a relative sporulation value >0.5 was estimated at temperatures ranging from 21°C to 30°C for the dematium clade, and from 24°C to 30°C for the truncatum clade. The curve for the graminicola clade had the lowest value of *c* (0.664), such that high sporulation for that clade occurred for temperatures between 17°C and 33°C.

### Within-clade variability in temperature responses

The distributions of residuals calculated as observed—estimated values are shown in Figure 4. Overall, most residuals were within an interval of ±0.5. Wider intervals were observed for infection and sporulation than for conidial germination and mycelial growth, and mycelial growth had the lowest variability. In all distributions, means of residuals were close to 0 with some asymmetry.

Residuals of mycelial growth were within an interval of ±0.25, with some outliers for four of the clades (Figure 4A). Greater variability, with higher numbers of outliers, was observed for acutatum, gloeosporioides, and orbiculare clades; within-clade variability seemed to increase with the number of species in a clade. Although the dematium clade included two species, data on mycelial growth were retrieved only for one of those species (*C. dematium*), leading to a distribution of residuals close to 0 (±0.07). The results suggest some differences in the dynamics of mycelial growth for species within the same clade.

For conidial germination, residual distributions were similar for all clades (±0.25), independent of the number of species in the clade, i.e., patterns of conidial germination were similar for all species in the same clade (Figure 4B). The only exception was in the orbiculare clade, which had greater variability (an interval of ±0.3) and a higher number of outliers than the other clades; much of the variability in the orbiculare clade was caused by *C. trifolii*, which had more restricted temperature requirements and a lower Topt than the other two species in the clade.

Variability was higher for infection than for the other biological processes (Figure 2), with residuals distributed in an interval of ±0.4; that was true for all clades and particularly for acutatum and gloeosporioides. In the acutatum clade, *C. godetiae* caused few infections when temperatures were <20°C or >25°C, resulting in high residuals between observed data and fit data for the clade. In the gloeosporioides clade, *C. musae* infected its host (banana) when temperatures were between 25°C and 32°C; however, other species in this clade also showed high infection at lower temperatures (15°C–24°C). Additional sources of variability for infection (other than the identity of the *Colletotrichum* species) were the identity of the host plant and the organs that were infected. There were 11 and 8 hosts for acutatum and gloeosporioides clades, respectively. In the acutatum clade, higher residuals were obtained for infections of leaves of coffee, celery, and azalea than for infections of both leaves and fruits of fruit trees like apples and olives. In the gloeosporioides clade, higher residuals were obtained for infections of fruits of banana and guava, and jointvetch plants than for fruits of apple and strawberry or for leaves of coffee. The effect of temperature on infection was similar for all the species in the orbiculare clade, with a residual interval of ±0.25 (Figure 4C). Variability was low in graminicola (±0.09) and destructivum (−0.06 to 0.08) clades.

Most of the residuals of sporulation were within an interval of ±0.4 (Figure 4D). Variability was highest for acutatum, destructivum, and gloeosporioides clades. Data on sporulation in the acutatum clade were available only for *C. acutatum* and *C. lupini*, which differed in their patterns of sporulation, especially at temperatures from 5°C to 15°C. Sporulation of *C. lentis* in the destructivum clade was higher than sporulation of *C. destructivum* when temperatures ranged between 15°C and 25°C. Variability in the gloeosporioides clade was mainly caused by *C. gossypii*, which did not sporulate at 12°C or 33°C, and by *C. fragarie*, which had high sporulation at about 30°C but low sporulation at 20°C and 35°C.

### Clusters for clades

**Figure 9:**
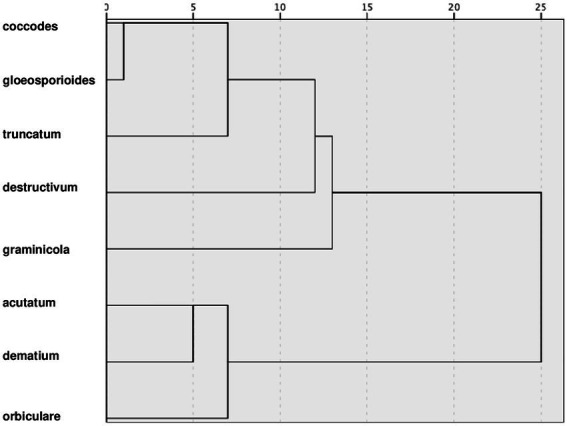
Dendrogram resulting from a hierarchical cluster analysis of the cardinal temperatures (minimum, optimum, and maximum) and values of parameter *a*, *b*, and *c* of Equation (1) for mycelial growth, conidial germination, conidial infection, and sporulation summarized in Table 2.

The hierarchical cluster analysis grouped the clusters at different rescaled distances (Figure 9); at the intermediate distance, four clusters were identified. The first cluster, which included coccodes, gloeosporioides, and truncatum clades, had optimum temperatures ranging between 25°C and 28°C for all biological processes; high minimum temperatures (5°C–12°C) for conidial germination, infection, and sporulation; and similar parameter values (Table 2). Compared to the first cluster, the fourth cluster (which included acutatum, dematium, and orbiculare clades) had lower optimum temperatures (21°C–25°C) and lower minimum temperatures (about 5°C). The fourth cluster also had similar within cluster values of *a*, *b*, and *c* for mycelial growth and conidial germination but dissimilar within cluster c values for conidial infection and sporulation in the case of the dematium clade. The second and third clusters contained one clade each, i.e., the destructivum clade and the graminicola clade, respectively. The destructivum clade had generally high values of *a* (except for infection by conidia), cardinal temperatures similar to those of the first cluster for conidia germination and sporulation, and lower cardinal temperatures than those of the other clusters for mycelial growth and infection. The graminicola clade had the highest minimum and optimum temperatures for conidial infection, which were 15°C and 30°C, respectively, and values for *a* that were similar to those in the destructivum clade, resulting in narrow, bell-shaped temperature-response curves (e.g., infection in Figure 2B).

## Discussion

Since the last century, several studies have investigated the temperature requirements of many *Colletotrichum* species causing anthracnose on cultivated plants. Here, we conducted systematic literature review was carried out to collect and analyze available information on the effect of temperature on the mycelial growth, conidial germination, infection by conidia, and sporulation of *Colletotrichum* species. The use of a systematic review enables researchers to locate and assemble what is known from the literature, and to synthesize the research findings into an accessible format (Mulrow, 1994). A systematic approach also reduces errors, limits research bias, and improves the communication of the information (Madden and Paul, 2011; Candel, 2014; Scherm et al., 2014). In the current study, we considered a total of 118 papers (from an initial number of 1,641) containing information on the effect of temperature on the four biological processes of 19 *Colletotrichum* species belonging to eight clades and on 27 host plants. Anthracnose pathogens have often been referred to as *C. gloeosporioides* or *C. acutatum* because the identification procedures often failed to include the use of multiple markers, and frequently, only the ITS region was analyzed (Zakaria, 2021). With the use of molecular phylogenetic analysis for identification and characterization of *Colletotrichum* species, diverse species have been reported to be associated with anthracnose diseases, and have been described and grouped into phylogenetic clades (Damm et al., 2009, 2012, 2013, 2014; Weir et al., 2012; Vieira et al., 2020; Talhinhas and Baroncelli, 2021). To our knowledge, the current report is the first to summarize the available published information with the aim of drawing robust conclusions regarding the similarities and differences in effects of temperature on *Colletotrichum* species grouped into phylogenetic clades.

Although *Colletotrichum* is one of the most studied genera of plant pathogens, important gaps about the effects of temperature on some biological processes for some species and clades still remain. Researchers have paid much more attention to the effects of temperature on mycelial growth than on conidial germination, infection, or sporulation. The studied temperature ranges were frequently restricted around the supposed optimum temperature. For example, conidial infection by *C. godetiae* and *C. fragariae* was tested between 10°C and 30°C (Smith and Black, 1987; López-Moral et al., 2019; Zhao et al., 2020), resulting in missing data on the minimum and maximum temperatures able to support infection. The most studied clades were the gloeosporioides and acutatum clades, which were also the clades with the largest number of species. In the acutatum clade, however, complete information was collected only for *C. acutatum* and *C. lupini*. Information was missing for infections caused by *C. nymphaea*, which is associated with olive and strawberry anthracnose (Antelmi et al., 2019; Wang et al., 2019). Specific experiments on the effects of temperature on sporulation have never been performed for *C. godetiae*, *C. nymphaea*, or *C. simmondsii*. We also could not find complete information for species in destructivum and dematium clades. This was unexpected, because species of these clades cause anthracnose in several legumes and ornamental plants (Smith et al., 1999; Tomioka et al., 2011, 2012), and because the gaps involve key aspects of the pathogen life cycle such as germination of conidia, infection, and sporulation. Overall, the insufficient database obtained through our literature review highlighted knowledge gaps on the temperature requirements for *Colletotrichum* spp. growth, infection, and sporulation that should be the foundation for the development of efficient disease control strategies.

Diverse species of *Colletotrichum* causing anthracnose are also of quarantine concern. For instance, *C. acutatum* is a quarantine pest in Israel and Tunisia, and is a regulated non-quarantine pest in several other countries (EPPO, 2022). In Europe, *C. gossypii* is well established in most of the cotton-growing areas; however, the pathogen is not known to occur in Greece where it is a potential quarantine pest (EPPO, 2022). These *Colletotrichum* species could spread to other areas where they would likely reduce crop yield and quality. It is therefore important to know the temperature requirements and other environmental conditions favorable for the development of *Colletotrichum* species in order to support pest risk assessment (Jeger et al., 2018; Bragard et al., 2021) and climate matching analysis regarding the introduction of anthracnose pathogens in new areas (Sutherst et al., 2011).

Equations developed in this work may help to identify similarities and differences in the effects of temperature on *Colletotrichum* species grouped into phylogenetic clades. High concordance (CCC ranging from 0.79 to 0.994) between observed and predicted data and low levels of residual errors (RMSE ≤0.2) indicated that our equations reliably represent the effect of temperature on the four biological processes. Distribution of residuals between observed and predicted values also indicated that the general response to temperature was similar among species in the same clade. These findings add to the results of previous publications, which stated that species in the same clade share similar behavior in terms of host infection and colonization (Damm et al., 2012; Weir et al., 2012; Zakaria, 2021). The plotting of equation parameter values in three-dimensional (*a*-*b*-*c*) space showed that the clades were located near each other for mycelial growth and conidial germination. Based on these results, we conclude that, regardless of the specific ability of species to grow and germinate, the shape of the mycelial growth and conidial germination response curves to temperature was similar among the clades. On the contrary, more dispersed distributions were observed for infection by conidia and sporulation. A higher inter- and intra-clade variability for these biological processes may reflect the importance of pathogen-host interactions in the successful establishment of the fungi in their hosts (Gan et al., 2016; Liang et al., 2018; Yan et al., 2018) and in the production of secondary inoculum (Jackson and Schisler, 1992; Mello et al., 2004; Kumara and Rawal, 2008).

Using a cluster analysis based on similarities in equation parameter values and cardinal temperatures, we identified four groups. These groups generally reflect the distribution of clades in clades in three-dimensional plots (Figure 3); some differences, however, should be recognized. The first group, consisting of coccodes, gloeosporioides, and truncatum clades was consistent in the three-dimensional plots, with the three clades closely distributed for all biological processes. The second and third groups, consisting only of destructivum and graminicola clades, respectively, were often self-grouped in the three-dimensional plots, indicating the dynamics of their biological processes differed greatly from the dynamics of the other clades. Although acutatum, dematium, and orbiculare clades were grouped together by cluster analysis, they were quite dispersed in the three-dimensional plots. In particular, higher values of parameter *c* (which determines the size of the bell-shaped curve described by the temperature-dependent equation) were obtained for both conidial infection and sporulation for the dematium clade. Nevertheless, the cardinal temperatures of these three clades were consistent and were generally lower than those of other groups.

For some of the species considered in this research, epidemiological models have been created to predict disease development (e.g., Dodd et al., 1991; Moral et al., 2012; Singh, 2020). Most of the models are simple and consider only one component of the pathogen life cycle, mainly conidial infection. Furthermore, many of these models have never been validated against independent data, i.e., model predictions have not been compared with real-world observations different from those used for model development (Rossi et al., 2010). Before these models are used in practical disease control, validation with real data obtained from different areas and years with different epidemiological conditions should be performed to assess model accuracy and robustness (Rossi et al., 2010). A mechanistic, weather-driven model was recently developed and validated for ripe rot of grapes caused by the *Colletotrichum* species (Ji et al., 2021). Several advantages have been previously reported for mechanistic models over empirical models, including a high explanatory ability and the possibility of easily incorporating information from previous and new experiments regarding pathogen biology and epidemiology (De Wolf and Isard, 2007; Rossi et al., 2015). The current report should help researchers to conduct further research on the biology and epidemiology of *Colletotrichum* species as well as to develop mechanistic models for those anthracnose diseases that currently lack such models. The current report may also provide a foundation for the development of a model that can be applied to multiple *Colletotrichum* spp. and to multiple anthracnose diseases based on clade similarities.

## Data availability statement

The original contributions presented in the study are included in the article/Supplementary material; further inquiries can be directed to the corresponding author.

## Author contributions

IS and TJ performed the systematic literature review. IS and VR performed the data analyses. All authors contributed to the writing of the manuscript. All authors contributed to the article and approved the submitted version.

## Funding

This research was funded by the LIFE Programme of the European Union-project LIFE AGRESTIC, grant number LIFE17 CCM/IT/000062.

## Conflict of interest

The authors declare that the research was conducted in the absence of any commercial or financial relationships that could be construed as a potential conflict of interest.

## Publisher’s note

All claims expressed in this article are solely those of the authors and do not necessarily represent those of their affiliated organizations, or those of the publisher, the editors and the reviewers. Any product that may be evaluated in this article, or claim that may be made by its manufacturer, is not guaranteed or endorsed by the publisher.
