# Supplementary material for: Temperature requirements of Colletotrichum spp. belonging to different clades
Source: Front Plant Sci. 2022 Jul 22;13:953760. doi: 10.3389/fpls.2022.953760 (PMC9354546; doi:10.3389/fpls.2022.953760)
Supplement: Supplementary file 1 [file Data_Sheet_1.docx]

Supplementary Material

**Supplementary Table 1.** Goodness-of-fit of equation (1) for each biological process and *Colletorichum* species in each clade.

| **Process** | **Clade** | **Species** | **R^2^** | **CCC** | **RMSE** | **CRM** |
| --- | --- | --- | --- | --- | --- | --- |
| **Mycelial growth** | acutatum | *C. acutatum* | 0.911 | 0.954 | 0.108 | 0.04 |
|  |  | *C. godetiae* | 0.868 | 0.936 | 0.127 | -0.044 |
|  |  | *C. lupini* | 0.956 | 0.973 | 0.089 | 0.09 |
|  |  | *C. nymphaea* | 0.851 | 0.925 | 0.137 | -0.068 |
|  |  | *C. simmondsii* | 0.901 | 0.953 | 0.11 | -0.008 |
|  | graminicola | *C. graminicola* | 0.929 | 0.968 | 0.094 | -0.003 |
|  | destructivum | *C. destructivum* | 0.72 | 0.844 | 0.21 | 0.105 |
|  |  | *C. lentis* | 0.978 | 0.986 | 0.065 | 0.012 |
|  | coccodes | *C. coccodes* | 0.869 | 0.933 | 0.143 | 0.055 |
|  | dematium | *C. dematium* | 0.986 | 0.994 | 0.04 | 0.008 |
|  | gloeosporioides | *C. gloeosporioides* | 0.928 | 0.965 | 0.092 | 0.013 |
|  |  | *C. fragariae* | 0.879 | 0.94 | 0.119 | 0.045 |
|  |  | *C. gossypii* | 0.885 | 0.939 | 0.123 | -0.013 |
|  |  | *C. musae* | 0.905 | 0.953 | 0.114 | 0.023 |
|  | truncatum | *C. capsici* | 0.992 | 0.996 | 0.033 | 0.004 |
|  | orbiculare | *C. orbiculare* | 0.92 | 0.958 | 0.102 | -0.061 |
|  |  | *C. trifolii* | 0.867 | 0.933 | 0.129 | 0.051 |
|  |  | *C. lindemuthianum* | 0.85 | 0.928 | 0.134 | -0.006 |
| **Conidial germination** | acutatum | *C. acutatum* | 0.855 | 0.923 | 0.131 | 0.041 |
|  |  | *C. nymphaea* | 0.546 | 0.717 | 0.256 | -0.186 |
|  | graminicola | *C. graminicola* | 0.954 | 0.979 | 0.09 | 0.002 |
|  | destructivum | *C. destructivum* | 0.902 | 0.952 | 0.113 | 0.062 |
|  | coccodes | *C. coccodes* | 0.911 | 0.958 | 0.106 | -0.017 |
|  | dematium | *C. dematium* | 0.942 | 0.974 | 0.087 | -0.007 |
|  | gloeosporioides | *C. gloeosporioides* | 0.904 | 0.952 | 0.114 | -0.015 |
|  |  | *C. fragariae* | 0.77 | 0.898 | 0.189 | -0.15 |
|  |  | *C. gossypii* | 0.88 | 0.938 | 0.136 | 0.099 |
|  |  | *C. musae* | 0.703 | 0.8 | 0.21 | 0.047 |
|  | truncatum | *C. capsici* | 0.93 | 0.961 | 0.104 | -0.083 |
|  | orbiculare | *C. orbiculare* | 0.86 | 0.913 | 0.165 | 0.144 |
|  |  | *C. trifolii* | 0.776 | 0.83 | 0.199 | 0.058 |
|  |  | *C. lindemuthianum* | 0.84 | 0.875 | 0.191 | -0.228 |
| **Conidial infection** | acutatum | *C. acutatum* | 0.856 | 0.928 | 0.144 | 0.075 |
|  |  | *C. godetiae* | 0.416 | 0.614 | 0.3 | -0.412 |
|  |  | *C. lupini* | 0.857 | 0.888 | 0.185 | 0.175 |
|  |  | *C. simmondsii* | 0.808 | 0.894 | 0.185 | -0.042 |
|  | graminicola | *C. graminicola* | 0.985 | 0.99 | 0.055 | -0.036 |
|  | destructivum | *C. lentis* | 0.984 | 0.993 | 0.047 | 0.001 |
|  | coccodes | *C. coccodes* | 0.929 | 0.968 | 0.091 | 0.002 |
|  | dematium | *C. dematium* | 0.97 | 0.973 | 0.098 | 0.107 |
|  |  | *C. spinaciae* | 0.826 | 0.92 | 0.147 | 0.008 |
|  | gloeosporioides | *C. gloeosporioides* | 0.902 | 0.951 | 0.118 | -0.058 |
|  |  | *C. fragariae* | 0.92 | 0.925 | 0.152 | 0.189 |
|  |  | *C. gossypii* | 0.831 | 0.896 | 0.16 | -0.013 |
|  |  | *C. musae* | 0.587 | 0.815 | 0.219 | -0.032 |
|  | truncatum | *C. capsici* | 0.891 | 0.946 | 0.128 | -0.042 |
|  | orbiculare | *C. orbiculare* | 0.813 | 0.902 | 0.16 | 0.065 |
|  |  | *C. trifolii* | 0.829 | 0.907 | 0.173 | 0.004 |
|  |  | *C. lindemuthianum* | 0.853 | 0.926 | 0.176 | -0.09 |
| **Sporulation** | acutatum | *C. acutatum* | 0.924 | 0.954 | 0.11 | -0.024 |
|  |  | *C. lupini* | 0.668 | 0.832 | 0.237 | -0.118 |
| ­ | graminicola | *C. graminicola* | 0.983 | 0.99 | 0.055 | -0.039 |
|  | Destructivum | *C. destructivum* | 0.804 | 0.853 | 0.183 | -0.322 |
|  |  | *C. lentis* | 0.829 | 0.512 | 0.305 | 0.273 |
|  | coccodes | *C. coccodes* | 0.918 | 0.96 | 0.117 | 0.054 |
|  | dematium | *C. dematium* | 0.994 | 0.993 | 0.039 | 0.148 |
|  | Gloeosporioides | *C. gloeosporioides* | 0.885 | 0.93 | 0.125 | 0.016 |
|  |  | *C. fragariae* | 0.771 | 0.894 | 0.172 | -0.059 |
|  |  | *C. gossypii* | 0.656 | 0.834 | 0.258 | -0.492 |
|  |  | *C. musae* | 0.859 | 0.939 | 0.115 | 0.022 |
|  | truncatum | *C. capsici* | 0.851 | 0.926 | 0.141 | 0.081 |
|  | orbiculare | *C. orbiculare* | 0.909 | 0.931 | 0.135 | -0.285 |
|  |  | *C. trifolii* | 0.812 | 0.85 | 0.236 | 0.254 |
|  |  | *C. lindemuthianum* | 0.968 | 0.977 | 0.09 | -0.133 |





**Supplementary Figure 1.** Mycelial growth for (A) acutatum, (B) graminicola, (C) destructivum, (D) coccodes, (E) dematium, (F) gloeosporioides, (G) truncatum, and (H) orbiculare clades. Symbols show the average mycelial growth in (A) ● *Colletotrichum acutatum*, ■ *C. godetiae*, ♦ *C. lupini*, ▲ *C. nymphaea, ○ C. simmondsii*; in (B) ● *C. graminicola*; in (C) ● *C. destructivum*, ■ *C. lentis*; in (D) ● *C. coccodes*; in (E) ● *C. dematium*; in (F) ● *C. gloeosporioides*, ■ *C. fragariae*, ♦ *C. gossypii*, ▲ *C. musae*; in (G) ● *C. capsici*; and in (H) ● *C. orbiculare*, ■ *C. trifolii*, ♦ *C.lindemuthianum*. The dotted lines show the fit of data using a bete equation (1); equation parameters for each clade are summarised in Table 3.





**Supplementary Figure 2.** Conidial germination for (A) acutatum, (B) graminicola, (C) destructivum, (D) coccodes, (E) dematium, (F) gloeosporioides, (G) truncatum, and (H) orbiculare clades. Symbols show the average conidial germination in (A) ● *Colletotrichum acutatum*, ▲ *C. nymphaea, ○ C. simmondsii*; in (B) ● *C. graminicola*; in (C) ● *C. destructivum*; in (D) ● *C. coccodes*; in (E) ● *C. dematium*; in (F) ● *C. gloeosporioides*, ■ *C. fragariae*, ♦ *C. gossypii*, ▲ *C. musae*; in (G) ● *C. capsici*; and in (H) ● *C. orbiculare*, ■ *C. trifolii*, ♦ *C.lindemuthianum*. The dotted lines show the fit of data using a bete equation (1); equation parameters for each clade are summarised in Table 3.





**Supplementary Figure 3.** Sporulation for (A) acutatum, (B) graminicola, (C) destructivum, (D) coccodes, (E) dematium, (F) gloeosporioides, (G) truncatum, and (H) orbiculare clades. Symbols show the average production of spores in (A) ● *Colletotrichum acutatum*, ♦ *C. lupini*; in (B) ● *C. graminicola*; in (C) ● *C. destructivum*, ■ *C. lentis*; in (D) ● *C. coccodes*; in (E) ● *C. dematium*; in (F) ● *C. gloeosporioides*, ■ *C. fragariae*, ♦ *C. gossypii*, ▲ *C. musae*; in (G) ● *C. capsici*; and in (H) ● *C. orbiculare*, ■ *C. trifolii*, ♦ *C.lindemuthianum*. The dotted lines show the fit of data using a bete equation (1); equation parameters for each clade are summarised in Table 3.
